# Draft Genome Sequence of the Plant Growth-Promoting Rhizobacterium *Pseudomonas fluorescens* Strain CREA-C16 Isolated from Pea (*Pisum sativum* L.) Rhizosphere

**DOI:** 10.1128/genomeA.01456-16

**Published:** 2017-01-26

**Authors:** Nunzio D’Agostino, Roberto Sorrentino, Riccardo Scotti, Melania Salzano, Vincenzo Aurilia, Massimo Zaccardelli

**Affiliations:** a CREA-ORT, Consiglio per la Ricerca in Agricoltura e l’Analisi dell’Economia Agraria, Centro di Ricerca per l’Orticoltura, Pontecagnano Faiano, Salerno, Italy; b National Research Council of Italy, Institute for Mediterranean Agriculture and Forest Systems, Ercolano, Naples, Italy

## Abstract

Herein, we report the draft genome sequence of *Pseudomonas fluorescens* strain CREA-C16, a plant growth-promoting rhizobacterium that was isolated from the rhizosphere of *Pisum sativum* L. plants. The genome sequence is ~6 Mb in size, with a G+C content of 60.1%, and includes 4,457 candidate protein-encoding genes.

## GENOME ANNOUNCEMENT

*Pseudomonas fluorescens* strain CREA-C16 was originally isolated from the rhizosphere of *Pisum sativum* L. plants cultivated in the Sele Valley (Campania, Italy) during 2007 and examined for its plant growth-promoting properties. The ability of this strain to promote root elongation and growth of tomato (*Solanum* *lycopersicum* L.) plants (72% increase in leaf dry weight compared to noninoculated plants), to release antifungal as well as phytohormone-like substances, and to produce fluorescent pigments makes it an efficient plant growth-promoting rhizobacterium (PGPR).

Furthermore, tomato plant roots are subjected to major transcriptome reprogramming after the early interaction with *P. fluorescens* strain CREA-C16. Genes encoding calmodulin, calmodulin-like proteins, and ethylene-responsive element binding factors are involved in plant root response to this PGPR.

Genomic DNA was extracted from a pellet of cells using the cetyltrimethylammonium bromide (CTAB) method, according to the protocol described by Ausbel et al. (1).

A draft genome sequence of *Pseudomonas fluorescens* strain CREA-C16 was generated from a paired-end library constructed with the Nextera library preparation kit. The sequencing (2 × 101 bp) was performed on a HiSeq 2500 device and generated 1.150 million reads (estimated insert size, ~1,100 bp). After filtering out low-quality reads, trimming of 10 bases from the 3′ end, and adapter removal, preprocessed reads were re-paired using the fastqCombinedPairedEnd.py script.

High-quality reads were *de novo* assembled with Velvet (2) into 132 contigs (k-mer size, 41) with minimum length of 200 bp, ranging from 204 bp to 415,383 bp and a *N*_50_ length of 110,307 bp.

Those contigs were ordered by Ragout version 0.2 (3) using *Pseudomonas fluorescens* Pf-0 (GenBank accession number CP000094.2) as the reference genome.

The assembly was further improved by running SOAP GapCloser version 1.12 (4), based on high-quality Illumina paired- and single-end reads. The result was a draft genome sequence of 5,758,958 bp, including gaps, with a G+C content of 60.1%. Per-base sequence coverage was calculated by mapping high-quality reads to the newly assembled genome using bowtie2 version 2.1.0 (5) and then running the coverage tool from the BEDtools package (6). The mean and median coverage depths were 23.24× and 26×, respectively.

Reference-based gene identification was performed running the prokaryotic gene finder tool EasyGene (7) using *Pseudomonas aeruginosa* PAO1 as reference, the PRODIGAL gene-finding algorithm (8), and the FgenesB (Softberry) using the “archae bacterial generic” as the closest organism. The final annotation includes only those genes predicted by at least two of three prediction tools. The annotation process resulted in 4,457 candidate protein-encoding genes.

Functional annotation was performed by BLAST search (e-value, < 10^-5^) of the predicted protein sequences against UniProtKB (9). Gene model descriptions were refined using InterProScan (10) for the identification of conserved protein domains and GO terms retrieval, and KAAS (11) was used for KEGG Orthology (KO) assignments and KEGG pathway mapping.

The annotation includes genes encoding enzymes involved in the indole-3-acetic acid (IAA) production from tryptophan, as well as a gene encoding the enzyme 1-aminocyclopropane-1-carboxylate (ACC) deaminase, a key factor in facilitating plant growth promotion.

### Accession number(s).

This whole-genome shotgun project has been deposited at DDBJ/EMBL/GenBank under the GenBank accession no. CP017951. The version described in this paper is the first version.
